# Comparison of Lures Loaded with Codlemone and Pear Ester for Capturing Codling Moths, *Cydia pomonella*, in Apple and Pear Orchards using Mating Disruption

**DOI:** 10.1673/031.010.13901

**Published:** 2010-08-23

**Authors:** D.E. Fernández, L. Cichón, S. Garrido, M. Ribes-Dasi, J. Avilla

**Affiliations:** ^1^ INTA Alto Valle. Ruta Nac. 22, Km 1190, General Roca, Río Negro, Argentina; ^2^ Centro UDL-IRTA de R+D, Universidad de Lleida, Rovira Roure 191, 25198, Lleida, Spain

**Keywords:** traps, kairomone, monitoring, female capture

## Abstract

Studies were conducted in apple, *Malus domestica* Borkhausen and pear, *Pyrus communis* L. (Rosales: Rosaceae), orchards to evaluate the attractiveness of grey halobutyl septa loaded with 1 (L2) and 10 (Mega) mg of codlemone, 8E, 10E-dodecadien-1-ol, 3 mg of pear ester, ethyl (E,Z)-2,4-decadienoate (DA2313), and 3 mg of pear ester plus 3 mg of codlemone (Combo) to adult codling moth, *Cydia pomonella* (L.) (Lepidoptera: Tortricidae). All studies were conducted in orchards treated with pheromone mating disruption. All four lures were tested on diamond-shaped sticky traps placed in 60 plots of apple and 40 plots of pears in 2003/04, and in 62 plots of apples and 30 of pears in 2004–05. Combo lures attracted significantly more moths (males + females) than all the others in both years. Comparisons among flights showed significant differences mainly for flight 1 and 2, but not always for flight 3. Mega lures provided no significant improvement compared with L2 lures during both seasons regarding the total number of moths. Combo and DA2313 lures attracted fewer females than males during the whole season. For most sample dates, more virgin than mated females were attracted to Combo lures, except during the third flight, and the overall ratio was 60:40, although the difference was not statistically significant. We conclude that the Combo lures are better indicators of codling moth activity in pheromone treated orchards, regardless of pest population level, when compared with similar lures containing codlemone or pear ester alone.

## Introduction

The codling moth, *Cydia pomonella* (L.) (Lepidoptera: Tortricidae), is an important pest of apple, *Malus domestica* Borkhausen, and pear, *Pyrus communis* L. (Rosales: Rosaceae) orchards worldwide. It is also the key pest in 40,000 ha of pome fruit orchards in the upper valley of Rio Negro and Neuquén, Argentina. In this area *C. pomonella* has three generations per year from September to March. A phenology model for the pest based on degree-days (DD) was implemented in 1989, based on a lower threshold of 10° C and a biofix start (August 1^st^). The first flight started at the end of September (70 DD), and ended in mid December (800 DD). The second flight occurred from mid December (750 DD) to mid February (1,300 DD), and the third flight overlaped the second one starting in February (1,200 DD) and ending in March (2,000 DD).

From its discovery and synthesis, the main component of the codling moth pheromone, 8*E*, 10*E*-dodecadien-1-ol, codlemone (Roelofs et al. 1971), has been widely used for monitoring (Howell and Quist 1980; Charmillot 1980; Riedl et al. 1986) and, during the last two decades, for implementing pheromone-based mating disruption control programs in many countries (Charmillot 1990; Gut and Brunner 1996; Bosch et al. 1998; Cichón and Fernández 1999; Witzgall et al. 2008). Mating disruption control strategies have two main constraints in order to be successful: the need for low population levels (Moffit and Westigard 1984; Vickers and Rothschild 1991), and the need for a reliable monitoring system (Gut and Brunner 1996).

High population levels can be reduced by management programs that include sanitation combined with the use of conventional or biological insecticides. However, monitoring a pheromone-treated orchard with traps baited with a pheromone lure leads to a generally not reliable monitoring system. Furthermore, damage risk depends mostly upon female activity and distribution, and male capture using codlemone only allows for an indirect estimation of female occurrence (Howell 1991). The discovery of new compounds that replace or complement codlemone and also attract codling moth females has been reported in recent years (Light et al. 2001; Hern and Dorn 2002; Ansebo et al. 2004; Coracini et al. 2004; Casado et al. 2006). One of them, known as “pear ester” [(2*E*, 4*Z*) ethyl decadienonate], is now available and is being used world-wide (Ioriatti et al. 2003; Il'ichev 2004; Knight et al. 2005a). In this study, we compared different lures loaded with codlemone, pear ester and a combination of both compounds, in order to assess their effectiveness in orchards treated with pheromone-based mating disruption.

## Materials and Methods

Studies were conducted in 2003/04 and 2004/05 in one hundred apple (‘Delicious’, ‘Granny Smith’, and ‘Gala’) and pear (‘Bartlett’, ‘D'Anjou, and Packham Triumph’) plots that totaled 200 ha. The plots were part of 15 contiguous orchards forming a rectangle of 2,000 m by 1,000 m. Previous harvest damage from codling moth in the test area averaged 4–6%. In 2003 these orchards started a Codling Moth Control Areawide Project (PAS) situated near Allen, Río Negro, Argentina and all were treated with hand-applied pheromone dispensers (Isomate C-Plus, Shin-Etsu, www.shinetsu.co.jp — 1,000/ha; NoMate CM, Scentry Biologicals, www.scentry.com — 1,000/ha; RAK CP, BASF, www.basf.com — 600/ha; CheckMate CM XL1000, Suterra, www.suterra.com — 600/ha) placed in the upper third of the canopy.

Diamond-shaped sticky traps (Pherocon® IIB, Trécé, Inc., www.trece.com) baited with grey halobutyl elastomer septa (Trécé, Inc.) were used in all tests to monitor codling moth densities. Lures were replaced in accordance with manufacturer guidelines (every 60 days) and liners were replaced after an accumulation of 30 moths was captured or as needed if the sticky surface was compromised. The mean heights of orchard canopies varied from 2.5 to 5.5 m, and traps were placed on wooden poles ≈0.5 m below the top of the canopy. Traps were checked weekly and all captured moth were counted, sexed, and the mating status of dissected females (presence or absence of spermatophore in the bursa copulatrix) was determined. Organophosphate and carbamate insecticides were applied at a regular basis (every 14 days) from mid October through harvest during 2003/04. During 2004/05, organophosphate insecticides were applied up to the end of the first flight (mid December) and a 2 moth/trap/week threshold for spraying was used through the remainder of the growing season.

**2003/04.** A study to compare four lure loadings (1 mg of codlemone — L2 lure; 10 mg of codlemone — Mega lure; 3 mg of pear ester — DA2313 lure; 3 mg of codlemone + 3 mg of pear ester — Combo lure); all prepared by Trécé, Inc., was conducted from 10 Oct 2003 to 30 Mar 2004. The lure comparison was replicated one hundred times, with each replicate covering one hectare and separated by at least 70 m from the next one. Treatments within each replicate were placed 25 m apart, in randomized, complete block. Overall the test area was a square-shaped. Sixty replicates were placed in apple and forty in pear plots.

**2004/05.** The 2003/04 study was repeated from 18 Oct 2004 to 30 Mar 2005 in the same test orchards. Ninety-two replicates were placed in 2004–2005, with sixty two replicates placed in apple and thirty in pears.

Unfortunately no 3 mg lures with codlemone were available commercially to be included in the test, but based on McNally and Barnes (1980) we assume that the 3 mg lures under the condition of the test should capture about the same number of moths as the 1 mg lure.

In both seasons each trap was georeferenced with a GPS unit (Garmin eTrex-Vista, Garmin International, Inc. www.garmin.com). All traps with codlemone and pear ester were serviced independently by different technicians in order to avoid cross-contamination. Technicians wore latex gloves when handling traps.

Damage assessments were done by randomly observing 1,000 fruits per hectare at the harvest of each cultivar. During 2003/04, 40,400 pears and 59,000 apples were evaluated, while 62,800 apples and 32,500 pears were evaluated 2004/05, and codling moth damage (presence or absence) was recorded. The damage was referred as percentage of affected fruit.

**Data analysis.** Data were analyzed separately for each season and aggregated by date. The effect of lure loading was analyzed through a Generalized Linear Model. The random variable showed a Poisson distribution (y ≈ P *oi* (λ) / µ = λ≥0) and the lineal predictor was *η* = α + τ_i_ + δ_j_ + (τδ)_ij_ + *βX*_k_ / *i* (lure) = 1,2,3,4; j (apple/pear) = 1,2; k (flight) = 1,2,3. The link function was the canonic (known as logarithmic in this type of distribution). A factorial arrangement for the expected average was proposed, where the considered factors were the above mentioned in the lineal predictor (lure, species, flight). The proportion of each sex in Combo and DA2313 lures within each flight was analyzed. The random variable showed a binomial distribution (male/female) and the lineal predictor was *η* = α + τ_i_ + δ_j_ + (τδ)_ij_ + *βX*_k_ / *i*. The link function was the canonical (logit). Within females a similar analysis was carried out in order to compare the proportion of virgin and mated females. The proportion of captured moths was analyzed within each species (pear/apple) in Combo and DA2313 lures in each flight. The random variable showed a binomial distribution (pear/apple) and the lineal predictor was *η* = α + τ_i_ + δ_j_ + (τδ)_ij_ + *βX*_k_ / *i*. The link function was the canonical (logit). In order to prove the goodness of fit of the models, the relationship between the deviance or the χ^2^ of Pearson and the degree of freedom from the corresponding model, was used. Since in all the analyzed cases this ratio was higher than 1, an over dispersion parameter was introduced to each model. Differences among factors were assessed through the Wald (W) statistic (Long 1997, Vaeth 1985) with a 5% significance level (p=0.05) (StatSoft 2001).

## Results and Discussion

### Comparison of different lures

**Figure 1.**
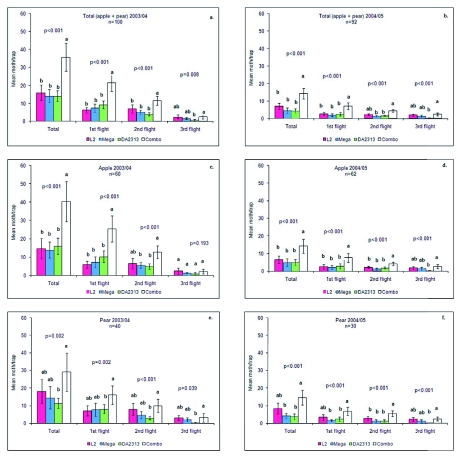
Mean (± SE) catches of Cydia pomonella in traps baited with L2, Mega, DA2313 and Combo lures in apple and pear orchards during 2003/04 and 2004/05. Means with the same letter for each flight are not significantly different using the Wald statistic with 5% significance level. High quality figures are available online.

The background codling moth population was high during the 2003/04 season, but very low during 2004/05 (Figures 1a, 1b). The success of the control measures carried out in 2003/04 clearly affected the 2004/05 population. This situation allowed the evaluation of lures under these two contrasting conditions. Significant differences among lures for the total number of codling moth catches per trap were found in most of the comparisons. In agreement with work done by Light et al. (2001), Knight and Light (2005), Knight et al. (2005b), lures with the combination of pear ester and codlemone (Combo) attracted significantly more moths (males + females) than the others in both years over the entire season (Figures 1a; 1b).

When lure data were analyzed among flights, the difference was significant for flights 1 and 2, but not for flight 3 (Figure 1a) during 2003/04, but was significant for all flights in 2004/05 (Figure 1b), showing a greater difference in a low-pressure situation. Similar results were found by Light et al. (2001) with Combo traps in apple orchards, where captures were high during the first flight and lower later in the season. Also in pears, the Combo lure outperformed all other lures for flight 1 and 2 (Figure 1e) even with low populations (2004/05) (Figure 1f). Overall, the performance of the L2 lure was similar to the Mega lure under these conditions. Higher catches with L2 lures by the end of the season could be due to a reduced release rate of mating disruption dispensers, since the response threshold of males is dose dependent (Witzgall et al. 2008) and also, high pheromone loads (10X) could have a reduced attraction under this scenario (McNally and Barnes 1980). Pear ester alone was comparable to L2 and Mega lures during the first and second flight, but attracted very few moths during the third flight (Figures 1b; 1c; 1d). Mega lures showed neither a significant improvement compared with L2 lures during both seasons regarding the total number of moth, nor improvement compared to DA2313 lures.

A false negative record occurs when, given a certain level of a population, no moths are captured; consequently no action is taken, leading to fruit damage. Given a certain population present in one area, it is assumed that all lures are able to detect this population. In consequence, all lures should register approximately the same number of positive (captures) and negative (zero) records, independently of the number of moth captured. Overall Combo lures had 1,085 negative records that were 26.9% less than DA2313 (1,459), 26.9% less than L2 (1,485) and 25.6% less than Mega (1,484) in 2003/04. In 2004/05 Combo lures had 1,605 negative records that were 17.3% less than DA2313 (1,933), 12.4% less than L2 (1,832) and 17.0% less than Mega (1,941). These data suggest that the Combo lure was more reliable to detect a given population of moths present in an area than all of the other lures.

### Comparison of apple vs. pears

**Figure 2.**
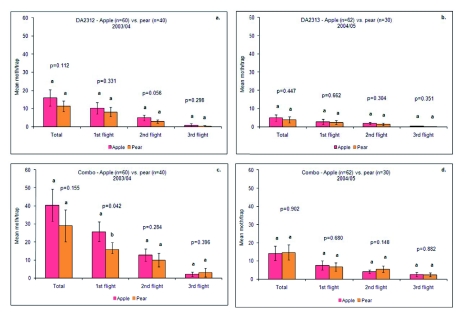
Mean (± SE) catches of Cydia pomonella in traps baited with DA2313 and Combo lures in apple and pear orchards during 2003/04 and 2004/05. Means with the same letter for each flight are not significantly different using the Wald statistic with 5% significance level. High quality figures are available online.

Light et al. (2001) reported that pear ester attraction was constant in walnut, low in pear and variable in apple orchards (high during the first flight and decreasing later in the season). Our results confirmed these findings for apple, but the same trend was also noted in pear. DA2313 and Combo lures both caught more moths in apple orchards than in pear orchards in both seasons, but these differences were not significant overall or for any of the individual flights (Figures 2a; 2b; 2c), except for the Combo lure in the first flight of 2003/04 (Figure 2c). Knight and Light (2001) and Il'ichev (2004) suggested that competition with orchard pear volatiles reduces the efficacy of pear ester later in the season, but our results do not support this hypothesis. However, the distribution and proximity of apple and pear plots and relative moth population, could account for the lack of the differences.

### Sex ratio

**Figure 3.**
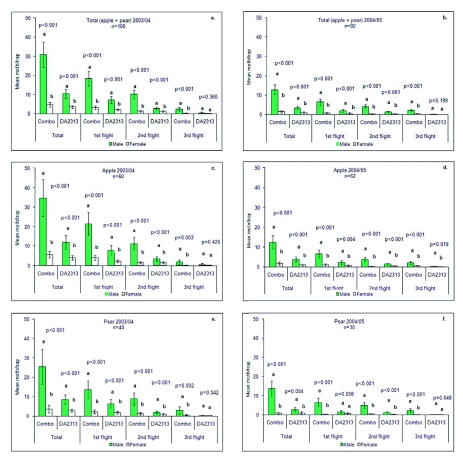
Mean (± SE) catches of male and female Cydia pomonella in traps baited with DA2313 and Combo lures in apple and pear orchards during 2003/04 and 2004/05. Means with the same letter for each flight are not significantly different using the Wald statistic with 5% significance level. High quality figures are available online.

Several previous studies have found that the pear ester attracted more males than females during the first flight of *C. pomonella*, and fewer males than females close to harvest (Light et al. 2001; Knight and Light 2005; Knight et al. 2005b). However, in this study we have confirmed findings by Il'ichev (2004) in Australia, and D. Bosch (Universitat de Lleida, Spain, personal communication) in Spain, that both Combo and DA2313 lures attracted fewer females than males during the whole season in apple and pear (Figures 3a; 3b; 3c; 3d; 3e; 3f).

Males attracted by Combo lures represented 86 to 89% of the total number of moths, while with DA2313 it was 74 to 76%. Also, Combo lures almost always attracted more males than DA2313 (Figure 3). Data suggest that there is a synergistic effect between the sex pheromone and the pear ester, towards attracting males. On the other hand, the number of females attracted by both lures in apple and pear was similar.

### Mated status

**Figure 4.**
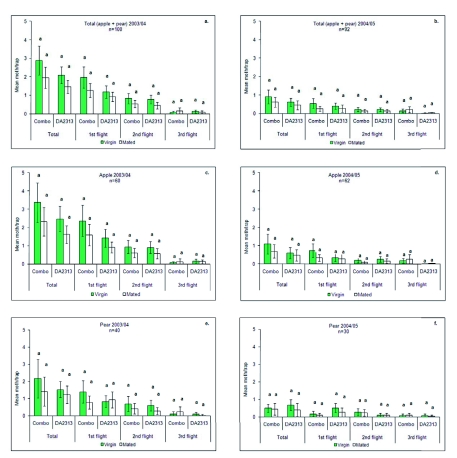
Mean (± SE) catches of virgin and mated female Cydia pomonella in traps baited with DA2313 and Combo lures in apple and pear orchards during 2003/04 and 2004/05. Means with the same letter for each flight are not significantly different using the Wald statistic with 5% significance level. High quality figures are available online.

Differences between virgin and mated females were not significant due mainly to high variation of the data and very low number of moths (Figures 4a; 4b; 4c; 4e), but the overall data show that 60% of the females captured were virgin and 40% mated. Combo lures showed a slight trend toward attracting more virgin females than DA2313, but due to the low number of females captured, significant differences were not found. Also DA2313 lures attracted slightly more mated females Unfortunately, due to the sanitation or “clean up” strategies applied to the whole area, it was not possible to relate the accumulated captures to damage, since the percentage of affected fruits at harvest was very low in both seasons (0.16% in 2003/04 and 0.19% in 2004/05). Most of the damage was found in apple plots, while pear damage was almost negligible in both seasons. Earlier harvest time in pear and other biotic and abiotic factors were likely to be the reason for these differences.

In 2004/05, moth densities were relatively low compared to 2003/04, however damage levels increased slightly. Standard spray programs were followed during the first generation, but subsequent sprays were based on an action threshold of 2 moths/trap/week. The use of the action thresholds may be a key factor producing the increase in damage, but this needs further analysis and a long-term study. We consider that most of the damage could be explained in some cases by poor spray application (timing and volume) and also could be related to immigration of moths from adjacent abandoned, or partially abandoned orchards. The increase in damage along orchard borders was reported by Brunner (2006) and Gut and Brunner (1998). These authors speculated that the increase in moth activity measured along orchard borders was a result of lower pheromone concentration in those areas.

### Final remarks

Even while some pest management professionals propose that the best monitoring system is the one that provides the most reliable estimate of the population, not necessarily the greatest number of insects, it is essential to have at least adequate numbers upon which to base a decision (Gut et al. 2004). This is important for the fruit industry in some countries like Argentina, which relies on export markets like Brazil where the codling moth is a quarantine pest. In this case, Combo lures should provide more reliable data upon which to base treatment decisions relative to other lures. However, even with the increase in codling moth captures noted with Combo lures, the improvement was still not enough to avoid the occurrence of false negative data (damage detection despite no trap captures). The occurrence of false negatives can be reduced by developing a better attractant, like the Combo lure, but also by proper trap and lure placement and maintenance. Traps, by themselves, are not reliable enough to completely replace the IPM scout, as visual observations by trained employees continues to remain a critical component to codling moth monitoring.

The new Combo lure technology represents an improvement over previously available monitoring systems; however several non-resolved issues should be addressed with future research. Trap densities, action thresholds, and the relevance of female capture data are important issues to investigate, as well as the interaction among plant volatiles and pheromones (Witzgall et al. 2008). Furthermore, female distribution, distance of flight, and other field behavior have been very difficult to measure due to the lack of effective attractants (Keil et al. 2001), but pear ester may facilitate these types of studies (Light et al. 2001).

We conclude that Combo lures performed better than the other three lures under this test situation, providing more reliable information about codling moth populations in pheromone-disrupted orchards.

## Abbreviations

Combo: pear ester plus codlemone;
ha: hectare
